# An Improved Protocol for Intact Chloroplasts and cpDNA Isolation in Conifers

**DOI:** 10.1371/journal.pone.0084792

**Published:** 2014-01-02

**Authors:** Leila do Nascimento Vieira, Helisson Faoro, Hugo Pacheco de Freitas Fraga, Marcelo Rogalski, Emanuel Maltempi de Souza, Fábio de Oliveira Pedrosa, Rubens Onofre Nodari, Miguel Pedro Guerra

**Affiliations:** 1 Departamento de Fitotecnia, Programa de Pós Graduação em Recursos Genéticos Vegetais, Universidade Federal de Santa Catarina, Florianópolis, Santa Catarina, Brazil; 2 Departamento de Bioquímica e Biologia Molecular, Núcleo de Fixação Biológica de Nitrogênio, Universidade Federal do Paraná, Curitiba, Paraná, Brazil; 3 Departamento de Biologia Vegetal, Universidade Federal de Viçosa, Viçosa, Minas Gerais, Brazil; University of California - Davis, United States of America

## Abstract

**Background:**

Performing chloroplast DNA (cpDNA) isolation is considered a major challenge among different plant groups, especially conifers. Isolating chloroplasts in conifers by such conventional methods as sucrose gradient and high salt has not been successful. So far, plastid genome sequencing protocols for conifer species have been based mainly on long-range PCR, which is known to be time-consuming and difficult to implement.

**Methodology/Principal Findings:**

We developed a protocol for cpDNA isolation using three different conifer families: *Araucaria angustifolia* and *Araucaria bidwilli* (Araucariaceae), *Podocarpus lambertii* (Podocarpaceae) and *Pinus patula* (Pinaceae). The present protocol is based on high salt isolation buffer followed by saline Percoll gradient. Combining these two strategies allowed enhanced chloroplast isolation, along with decreased contamination caused by polysaccharides, polyphenols, proteins, and nuclear DNA in cpDNA. Microscopy images confirmed the presence of intact chloroplasts in high abundance. This method was applied to cpDNA isolation and subsequent sequencing by Illumina MiSeq (2×250 bp), using only 50 ng of cpDNA. Reference-guided chloroplast genome mapping showed that high average coverage was achieved for all evaluated species: 24.63 for *A. angustifolia*, 135.97 for *A. bidwilli*, 1196.10 for *P. lambertii*, and 64.68 for *P. patula*.

**Conclusion:**

Results show that this improved protocol is suitable for enhanced quality and yield of chloroplasts and cpDNA isolation from conifers, providing a useful tool for studies that require isolated chloroplasts and/or whole cpDNA sequences.

## Introduction

The chloroplast genome of land plants usually harbors a conserved set of approximately 120 genes in a 120–160 kb pair genome, out of a genome of some 3,200 genes present in their cyanobacterial ancestor [1]. Land plant plastomes are mostly conserved and present little variation in size and gene content, ranging from 70,028 nucleotides and 25 protein coding genes in the nonphotosynthetic parasitic plant, *Epifagus virginiana* [2], to 217,942 nucleotides and 131 protein coding genes in *Pelargonium*×*Hortorum* [3]. Although chloroplast genomes contain highly conserved essential genes for plant growth and development, they also contain variable regions, i.e., intergenic regions and structural variations. In addition, they contain one of the few sets of characters that can transcend the life history of green plants and, hence, generate important evolutionary information. Therefore, chloroplast genome sequences can be used for comparative evolutionary studies within and between different groups of plants [4], [5], [6], as demonstrated by several works [7], [8], [9], [10], [11]. Furthermore, chloroplast genome sequences have been used to investigate gene function [12], [13], and they have been targeted for biotechnological applications [14], [15], [16], [17]. Based on the importance of land plant chloroplast DNA (cpDNA) in plant genetics, evolution and biotechnology, it has been a target in many plant genome sequencing projects [18], [19], [20]. To date, complete cpDNAs of more than 300 plants have been sequenced (ncbi.nlm.nih.gov/genomes/GenomesGroup.cgi?taxid = 2759&opt = plastid).

With rapid progress in sequencing technologies, chloroplast genome sequencing can be realized quickly as a result of small size and structural simplicity when compared to nuclear genomes. However, chloroplast genome sequences have been determined for only a very few families belonging to gymnosperms [18], [20], [21]. Especially for conifers, chloroplast genome sequences are available for families that include Cephalotaxaceae [11], Cupressaceae [19], Pinaceae [20], [22], [23], Podocarpaceae (database accession no. NC_020361.1) and Taxaceae (database accession no. NC_020321.1), but not the Araucariaceae family.

Chloroplast DNA isolation has been a major challenge, hindering widespread applications in different plant groups. Chloroplast isolation in conifers by such conventional methods as sucrose gradient [24] and high salt [25] has, thus far, not been successful. This most likely results from the high volume of contaminants, including polyphenols, oleoresins, terpenoids and polysaccharides, present in conifer needles, making difficult the acquisition of intact isolated chloroplasts and high quality cpDNA [26]. For conifers, whole cpDNA sequencing protocols have been based on total DNA isolation, followed by cpDNA fragments amplification by use of polymerase chain reaction (PCR) with degenerate primers [10], [11], [20], [23]. However, this strategy is known to be time-consuming and difficult to implement because of differences in gene organization among different plant species [27] and “promiscuous” cpDNA present in the nucleus and mitochondrial genome [28], [29], [30], [31].

Therefore, the overall aim of the present work was to develop an efficient protocol for chloroplast isolation and subsequent high quality cpDNA extraction in conifers, using three different conifer families: *Araucaria angustifolia* and *Araucaria bidwilli* (Araucariaceae), *Podocarpus lambertii* (Podocarpaceae) and *Pinus patula* (Pinaceae).

## Materials and Methods

### Plant Material

Local *A. angustifolia* and *P. patula* seeds were purchased and germinated in the greenhouse of Federal University of Santa Catarina, Brazil. Needles were collected from 6 months plants; this procedure does not require authorization. *P. lambertii* young plants (n = 10) were collected at a private area, located at Lages, Santa Catarina, Brazil (27° 48′ 57′′ S, 50° 19′ 33′′ W), where the species is abundant, with the previous owner permission (José Antônio Ribas Ribeiro). This species are not considered under threat. After, the young plants were transplanted to greenhouse and maintained under this condition until the collection of needles. *A. bidwilli* young needles were collected at Botanical Garden, authorized by Federal University of Santa Catarina, Brazil. For each plant species, 25 g of fresh young needles were collected and stored in 4°C refrigerator for further chloroplast extractions.

### Protocols

**Figure 1:**
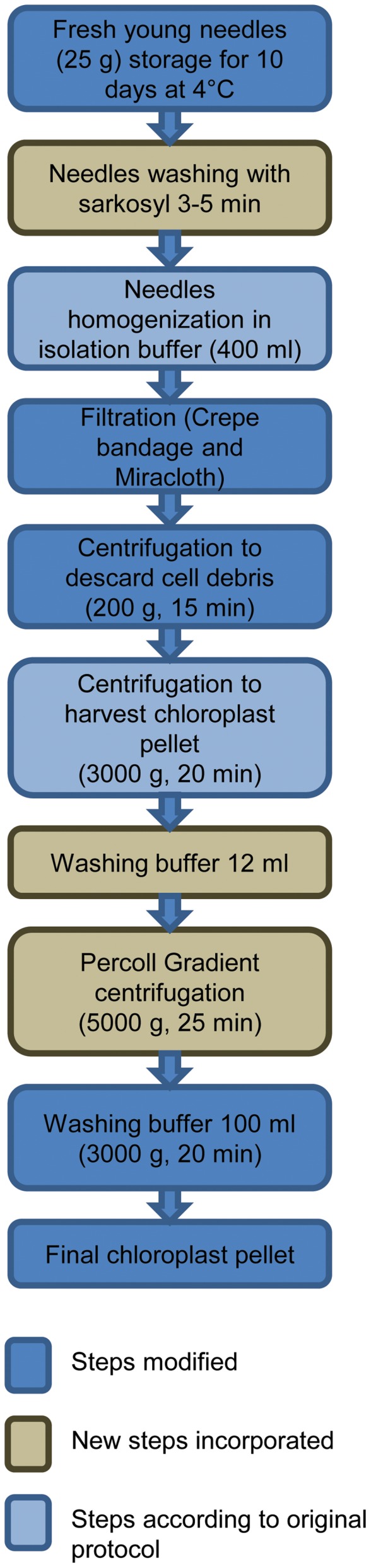
Flowchart showing the major steps for chloroplast isolation according to high salt plus saline Percoll.

The three chloroplast DNA isolation methods used here are described as follows:

**A) High salt plus saline Percoll gradient method (** **Figure 1** **).** All the following steps were carried out at 0°C, if not otherwise stated.

1. Prior to extraction, 25 g (fresh weight) of young needles were collected and kept in dark for 10 days at 4°C to decrease starch and resin level. Fresh needles were cleaned with 0.5% sarkosyl (Fluka, Ronkonkoma, NY) for 5 min to reduce microbial contamination and then washed 4 times with distilled water.
2. Needles were homogenized in 400 ml ice-cold isolation buffer (Table 1) for 30 s in a pre-chilled blender. Homogenate was filtered primarily into two layers of gauze bandage and then filtered again using two layers of Miracloth by softly squeezing the cloth.
3. Homogenate was centrifuged at 200 g for 15 min at 4°C. The nucleus pellet and cell-wall debris were discarded. The supernatant included chloroplasts suspended in it.
4. The supernatant was centrifuged at the higher centrifugal force of 3000 g for 20 min at 4°C, resulting in a chloroplast pellet with some contamination.
5. The pellet was gently resuspended in 12 ml of wash buffer (Table 1) using a paintbrush.
6. Homogenate was divided into 6 tubes (50 ml), each containing 20 ml Percoll (GE Healthcare, Uppsala, Sweden) gradient (70%–30%) and then centrifuged at 5000 g for 25 min at 4°C. The interface 70%–30% containing chloroplasts was collected.
7. Collected interface containing chloroplasts was washed twice with 100 ml of wash buffer and centrifuged at 3000 g for 20 min at 4°C to obtain the purified chloroplast pellet.

**Table 1:** Composition of chloroplast isolation buffers and wash buffers for modified high salt method, high salt plus saline Percoll method and sucrose gradient method.

High salt plus saline Percoll method	Modified high salt method [32]	Sucrose gradient [33]
Isolation Buffer (pH 3.8)	Isolation Buffer (pH 3.8)	Isolation Buffer
1.25 M NaCl	1.25 M NaCl	50 mM Tris-HCl (pH 8.0)
0.25 M ascorbic acid	0.25 M ascorbic acid	0.35 M sorbitol
10 mM sodium metabisulfite	10 mM sodium metabisulfite	7 mM EDTA
0.0125 M Borax	0.0125 M Borax	0.1% 2-mercaptoethanol
50 mM Tris-HCl (pH 8.0)	50 mM Tris-HCl (pH 8.0)	0.1% BSA
7 mM EDTA	7 mM EDTA	
1% PVP-40 (w/v)	1% PVP-40 (w/v)	
0.1% BSA (w/v)	0.1% BSA (w/v)	
	1 mM DTT	
**Wash Buffer (pH 8.0)**	**Wash Buffer (pH 8.0)**	**Wash Buffer**
1.25 M NaCl	1.25 M NaCl	50 mM Tris-HCl (pH 8.0)
0.0125 M Borax	0.0125 M Borax	0.35 M sorbitol
50 mM Tris-HCl (pH 8.0)	50 mM Tris-HCl (pH 8.0)	25 mM EDTA
25 mM EDTA	25 mM EDTA	
1% PVP-40 (w/v)	1% PVP-40 (w/v)	
0.1% BSA (w/v)	0.1% BSA (w/v)	
	1 mM DTT	

Both BSA and DTT were added just before the start of the experiment.

Percoll gradient solutions consisted of wash buffer with Percoll at a final concentration of 70% (v/v) and 30% (v/v).

Sucrose gradient solutions consisted of 50 mM Tris-HCl (pH 8.0), 25 mM EDTA and sucrose addition for a final concentration of 52% sucrose (w/v) and 30% (w/v) sucrose.

**B) Modified high salt method** [**32**] **.** All the following steps were carried out at 0°C, if not otherwise stated.

1. Prior to extraction, 25 g (fresh weight) of young needles were collected and kept in dark for 72 h at 4°C to decrease starch level stored in the needles. Fresh needles were cleaned with distilled water.
2. Needles were homogenized in 400 ml of isolation buffer (Table 1) for 30 s. Homogenate was filtered into centrifuge bottles, using two layers of Miracloth (Calbiochem, San Diego, CA) by softly squeezing the cloth.
3. The homogenate was centrifuged twice at 200 g for 20 min at 4°C. The nucleus pellet and cell-wall debris were discarded. Supernatant included chloroplasts suspended in it.
4. The supernatant was submitted to a higher centrifugal force (3500 g) for 20 min at 4°C, resulting in a chloroplast pellet contaminated with some nuclear DNA.
5. The pellet was gently resuspended in 250 ml of wash buffer (Table 1), using a paintbrush to wash the nuclear DNA attached to the chloroplast membrane, followed by centrifugation at 3500 g for 20 min at 4°C. The supernatant was discarded.
6. The pellet was resuspended again with 250 ml wash buffer and centrifuged at 3500 g for 20 min at 4°C to obtain the final chloroplast pellet.

**C) Sucrose gradient method** [**33**] **.**

1. Prior to extraction, about 25 g (fresh weight) of young needles were collected and kept in dark for 72 h at 4°C in order to decrease the starch level stored in the leaves. Fresh needles were cleaned with distilled water.
2. Needles were homogenized in 400 ml of ice-cold isolation buffer (Table 1) for 30 s. The homogenate was filtered into centrifuge bottles using two layers of Miracloth by softly squeezing the cloth.
3. The homogenate was centrifuged at 200 g for 15 min at 4°C. The nucleus pellet and cell-wall debris were discarded. The supernatant included chloroplasts suspended in it.
4. The supernatant was centrifuged at a higher centrifugal force (2000 g) for 20 min at 4°C, and the resulting chloroplast pellet showed some contamination.
5. The pellet was resuspended in 7 ml of ice-cold wash buffer (Table 1), using a soft paintbrush.
6. The homogenate was gently loaded into 6 tubes (50 ml) containing sucrose step gradient consisting of 18 ml of 52% sucrose and overlaid with 7 ml of 30% sucrose.
7. Step gradients were centrifuged at 3500 g for 60 min at 4°C.
8. The band from the 30–52% interface containing chloroplasts was collected, diluted twice with 200 ml of wash buffer, and centrifuged at 1500 g for 15 min at 4°C to gain the purified chloroplast pellet.

### Chloroplast DNA Isolation

Chloroplast DNA isolation was the same for all chloroplast pellets obtained using the three different isolation methods. DNA isolation buffer consisted of 100 mM NaCl, 100 mM Tris-HCl (pH 8.0), 50 mM EDTA, and 1 mM DTT.

1. The chloroplast lyse was obtained by incubating the chloroplast pellet with 8 ml of DNA isolation buffer, 1.5 ml 20% SDS, 20 µl 2-Mercaptoethanol and 30 µl Proteinase K (10 mg/ml) into a centrifuge tube at 55°C for 4 h.
2. The centrifuge tube was incubated on ice for 5 min, and then 1.5 ml 5 M KAc (pH 5.2) was added to the lyse mixture and chilled for more than 30 min. After that, the tube was centrifuged at 10000 g for 15 min at 4°C, and the pellet was discarded.
3. The supernatant was extracted with an equal volume of saturated phenol and chloroform:isoamyl-alcohol (24∶1) and centrifuged twice at 10000 g for 20 min.
4. An equal volume of isopropyl alcohol (about 10 ml) was added to the upper aqueous phase and incubated at −20°C overnight.
5. To obtain the DNA pellet, the tube was centrifuged at 10000 g for 20 min at 4°C. The cpDNA pellet was washed with 70% and 96% ethanol, air dried, and redissolved in 50 µl TE buffer.
6. The cpDNA samples were treated with RNAse, and the DNA band was visualized on a 0.7% agarose gel.
7. DNA purity and concentration were evaluated with Nanodrop®, based on 260/280 and 260/230 ratios.

### Microscopy Analysis

The integrity of isolated chloroplasts was assessed with a phase-contrast light microscopy using an inverted Olympus IX81 microscope [34]. Intact chloroplasts were considered those with pale yellow-green color and refractive, with a bright halo appearance around each plastid, whereas broken chloroplasts were those with a dark green, granular, and non-refractive appearance [34].

### Chloroplast Genome Sequencing

*A. angustifolia*, *A. bidwilli*, *P. patula* and *P. lambertii* cpDNAs were isolated using the high salt plus Percoll method. For each species, approximately 50 ng of DNA were prepared with the Nextera DNA Sample Prep Kit (Illumina, San Diego, USA) according to the manufacturer’s instructions. Chloroplast DNAs were sequenced using Illumina MiSeq (2×250 read length) at the Federal University of Paraná - Brazil. The obtained paired-end reads were assembled to reference genome sequence and estimate genome coverage, using the CLC Genomics Workbench 5.5 software. The reference chloroplast genome sequences of *Podocarpus totara* (NC_020361.1) and *Pinus thunbergii* (NC_001631.1) were downloaded from GenBank.

## Results and Discussion

### Chloroplast Isolation

Chloroplast isolation protocols are generally based on methods that employ high salt concentration buffers [32], sucrose density gradient [33], high salt buffers followed by sucrose gradient [35] and high sorbitol concentration buffers followed by Percoll gradient [36]. As the purity of intact chloroplasts is one of the critical steps of whole sequencing, a previous paper [19] considered the use of sucrose density gradients as the best method for separating nuclear DNA contamination from cpDNA. The present protocol is based on a high salt isolation buffer followed by saline Percoll gradient. The combination of these two strategies provided two advantages: better isolation of chloroplasts by use of the Percoll gradient and decreased contamination by polysaccharides, polyphenols, and proteins.

The first significant change in isolation protocol was the increase in storage time to 10 days at 4°C prior to extraction. This change led to a significant reduction in the viscosity of extraction buffer, possibly caused by a decrease in the polysaccharides and oleoresin concentrations. A similar strategy has been used in other protocols, in which 48–72 h at 4°C was enough to reduce the stored polysaccharides [32], [33]. However, because of the high amount of oleoresins and thick outer periclinal walls in conifers [37], [38], a longer time is required to reduce the concentration of these compounds.

Subsequently, needles were washed with sarkosyl, reducing material contamination. At the time of homogenization, it was observed that the high salt buffer was also responsible for the decrease in viscosity of the solution. The viscosity normally found in the homogenate (conifer needles and isolation buffer) is related to the high amount of resins and polysaccharides present in conifer needles, and its reduction enables faster and more efficient filtration, with lower material loss. In a previous paper [39], it was observed that the use of high salt buffers for DNA extraction increased the quality and yield of DNA extracted from plant tissues rich in polysaccharides.

Similarly, the initial centrifugation step, performed to pellet cellular debris was reduced to 200 g. We also reduced the initial centrifugation step at 200 g to only one centrifugation, while in the modified high salt method [32], it was performed twice. This reduction increased chloroplast yield and did not entail any reduction in the quality of isolated chloroplast as a result of the Percoll gradient step. The major steps for chloroplast isolation using the high salt plus Percoll method, including original, new and modified steps, are summarized in Figure 1.

**Figure 2:**
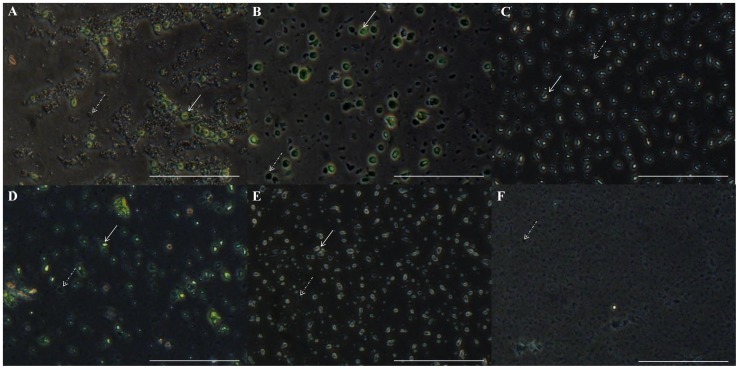
Chloroplast visualization of *Araucaria angustifolia* in phase contrast microscopy. (A) Chloroplasts isolated with improved high salt method; (B) Chloroplasts isolated with sucrose method; (C) Chloroplasts isolated with high salt plus Percoll method; (D–F) Micrographs during chloroplast isolation with high salt plus saline Percoll method; (D) Broken and intact chloroplasts before Percoll gradient centrifugation; (E) Intact isolated chloroplasts in interface 70/30% after Percoll gradient centrifugation; (F) Broken chloroplasts in upper 30% phase after Percoll gradient centrifugation. Dotted arrows indicate broken chloroplasts. Solid arrows indicate intact chloroplasts. Bar –50 µM.

Microscopy images showed the presence of some intact chloroplasts and a large amount of broken chloroplasts and other cellular debris in extraction when using the modified high salt and sucrose methods (Fig. 2A, B). On the other hand, the high salt plus saline Percoll method resulted in the presence of abundant intact chloroplasts (Fig. 2C).

Aiming to better characterize the efficiency of Percoll gradient, microscopy analysis was performed prior to the Percoll gradient centrifugation step, and the presence of both intact and ruptured chloroplasts could be observed (Fig. 2D). However, after centrifugation in Percoll gradient, the 70%/30% interface (Fig. 2E) contains abundant intact chloroplasts and only a few broken chloroplasts, while the upper phase contains many broken chloroplasts (Fig. 2F). In addition, below the 70% gradient, the formation of a white colored pellet composed of polysaccharides and other contaminants could be seen. Despite the high purity of chloroplasts observed immediately after Percoll gradient centrifugation, two subsequent centrifugations are essential to remove any residue of Percoll. The isolation of cpDNA without performing these two washes would be greatly affected by its presence. It is noteworthy that changes in the protocol enabled the isolation of the best quality chloroplasts, without the need of ultracentrifugation, which could be a limiting point in the procedure.

In addition to facilitating whole cpDNA sequencing, isolation of intact chloroplasts can also be applied in plastid proteome characterization studies. Comparative proteomics in *Triticum aestivum* and *Arabidopsis thaliana* chloroplasts have been recently developed using intact isolated chloroplasts. The results demonstrated that the quality of chloroplast isolation is a fundamental step of complete proteome characterization [40], [41].

### cpDNA Isolation

**Figure 3:**
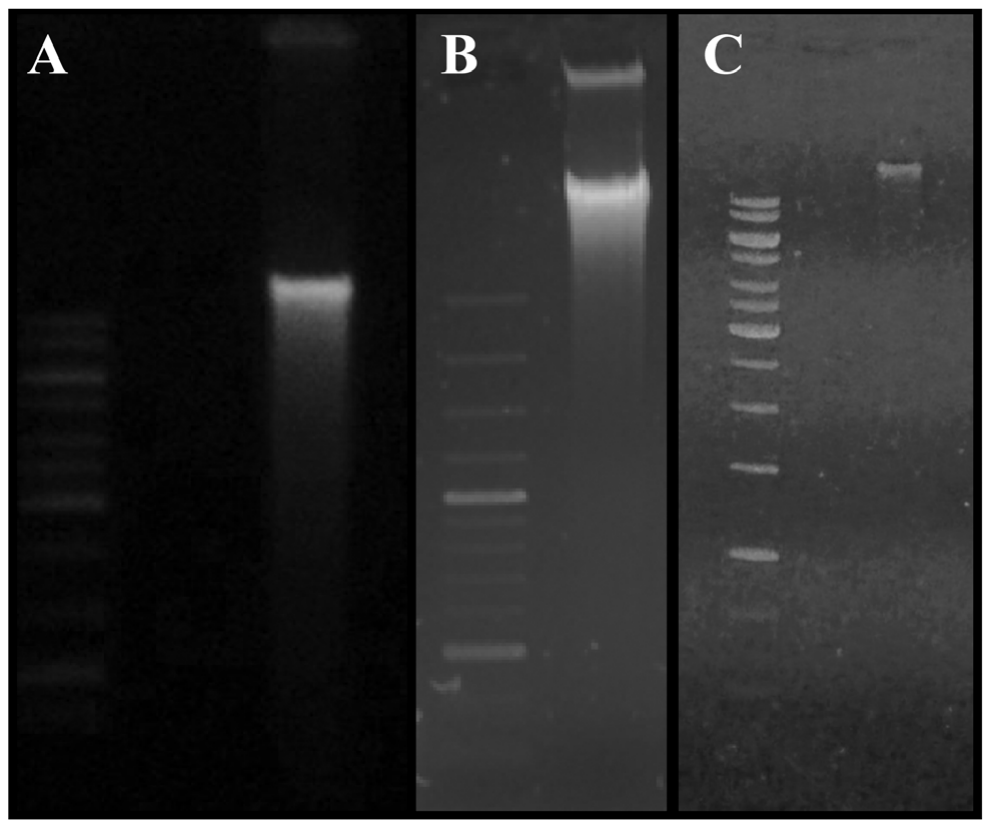
Chloroplast DNA visualization of *Araucaria angustifolia* in 0.7% agarose gel stained with ethidium bromide. (A) Ladder 1 kb and cpDNA isolated with modified high salt method; (B) Ladder 1 kb and cpDNA isolated with sucrose method; (C) Ladder 1 kb and cpDNA isolated with high salt plus saline Percoll method.

We also obtained isolated cpDNA with better quality and yield using this high salt plus saline Percoll method. Using the modified high salt and sucrose methods, bands in agarose gel revealed the presence of degraded DNA, indicating contamination with nuclear DNA and polysaccharides (Fig. 3A, B, respectively), while isolated cpDNA formed a well-defined band, which is indicative of high purity and polysaccharide-free cpDNA (Fig. 3C).

In addition, Nanodrop evaluation indicated higher cpDNA yield with the high salt plus saline Percoll method, about 3 times higher when compared to high salt methods and almost 9 times higher when compared to the sucrose method. Enhanced 260/280 and 260/230 ratios were observed in the high salt plus saline Percoll method, 2.05 and 1.99, respectively (Table 2). These ratios indicate a high purity of isolated cpDNA, which is a prerequisite for whole chloroplast sequencing. In the two other methods evaluated, contamination was observed with polyphenols and polysaccharides (Table 2). Moreover, when we used the sucrose method, a highly contaminated and oxidized DNA pellet was obtained. Taken together, we considered the high salt plus saline Percoll protocol as having the best yield and quality for cpDNA isolation from *A. angustifolia*. Thus, this method was applied to cpDNA isolation of *A. bidwilli*, *P. patula* and *P. lambertii*. As expected, a high quality in cpDNA isolated from all evaluated species was realized at 260/280>1.95 and 260/230>1.74 ratios (Table 3). All species showed cpDNA yield similar to *A. angustifolia,* with the exception of *P. lambertii.* However, even its cpDNA yield was sufficient for sequencing (Table 3).

**Table 2:** cpDNA from different isolation methods in *Araucaria angustifolia* sample.

Isolation Method	DNA concentration (ng/µl)	260/280	260/230
Modified high salt method	975.6	1.66	0.92
High Salt plus saline Percoll method	3438.3	2.05	1.99
Sucrose Gradient Method	472.4	1.52	0.47

Ratios evaluated with Nanodrop®, in a final volume of 40 µl.

**Table 3:** cpDNA ratios of selected conifers evaluated using Nanodrop®.

Plant species	DNA concentration(ng/µl)	260/280	260/230
*Araucaria angustifolia*	3438.3	2.05	1.99
*Araucaria bidwilli*	3038.0	1.95	1.74
*Podocarpus lambertii*	430.6	2.01	1.89
*Pinus patula*	1799.5	2.05	2.10

Samples were isolated with the high salt plus Percoll method in different conifer species. Final volume of 40 µl.

### Chloroplast Genome Sequencing

Improving technologies have made DNA sequencing faster, more accurate and far cheaper, creating opportunities to sequence the whole chloroplast genome in order to perform evolutionary and phylogenomic studies. To test the quality of cpDNA isolated by our new method, we sequenced the plastid genome of four conifer species (*A. angustifolia*, *A. bidwilli*, *P. patula* and *P. lambertii*) using the Illumina sequencing technology.

**Figure 4:**
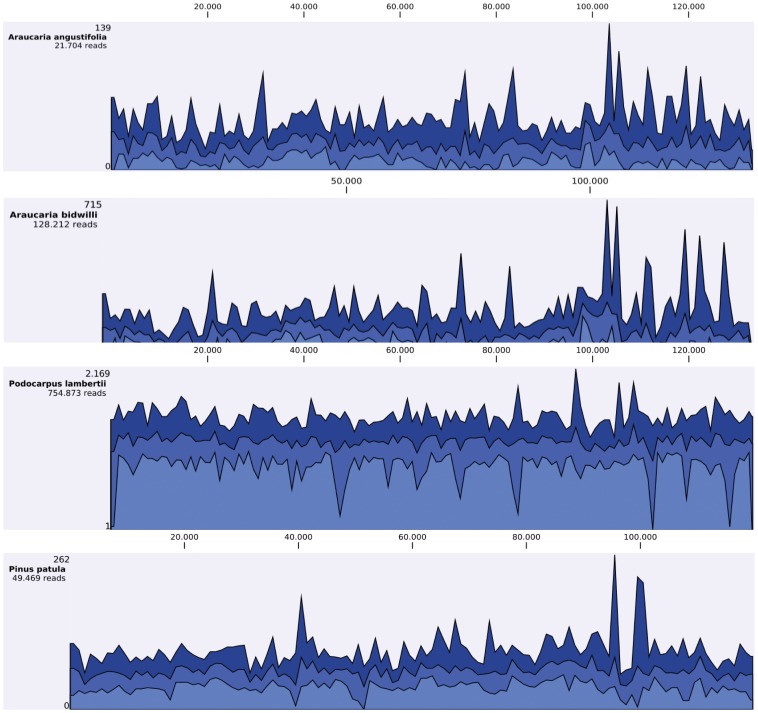
Reference graph track showing observed coverage values. Different colors show the minimum (light blue), mean (blue), and maximum (dark blue) observed coverage values for all genomic regions (data aggregation above 100 bp). *Araucaria angustifolia*, *Araucaria bidwilli*, and *Podocarpus lambertii* sequence reads were mapped on *Podocarpus totara*; *Pinus patula* sequence reads were mapped on *Pinus thunbergii*.

To estimate the efficiency of chloroplast genome sequence assembly with our cpDNA isolation protocol, we sequenced these four chloroplast genomes by using MiSeq Illumina sequencing with only 50 ng of cpDNA. In other sequencing protocols, about 5–10 µg [32], [33] were used for sequencing, thereby increasing the amount of plant material required for isolation and often limiting the use of the technique. A reference-guided chloroplast genome mapping was performed to estimate the genome average coverage (Fig. 4). The cpDNA sequencing generated a high average coverage for all species evaluated: 24.63 for *A. angustifolia*, 135.97 for *A. bidwilli*, 1,196.10 for *P. lambertii*, and 64.68 for *P. patula* (Table 4). Thus, in this study, all of the reference genomes were sufficiently covered for assembly.

**Table 4:** Average coverage of cpDNA evaluated from selected conifers with CLC Genomics Workbench 5.5 software.

Plant Species	Average Coverage	Reference Genome
*Araucaria angustifolia*	24.63	*Podocarpus totara*
*Araucaria bidwilli*	135.97	*Podocarpus totara*
*Podocarpus lambertii*	1196.10	*Podocarpus totara*
*Pinus patula*	64.68	*Pinus thunbergii*

cpDNA reads were mapped to reference genomes.

This protocol presents higher genome coverage when compared to protocols recently applied to conifers chloroplast genome sequencing, as those using total DNA followed by PCR amplification with degenerated primers that resulted in genome coverage only about 8-fold [11], [20]. Furthermore, this strategy is time-consuming and difficult to implement because of differences in gene organization among different plant species. *Cryptomeria japonica* cp genome was sequenced using sucrose gradient method, followed by DNA isolation with phenol/chloroform, DNA purification with DNeasy Plant Mini Kit (QIAGEN) and ATP-dependent DNase (TOYOBO) [19]. As shown in the present work, the protocol based on saline buffer followed by Percoll gradient results in higher quality DNA than sucrose gradient. Moreover, all these purification steps applied to the isolated DNA, such as the utilization of ATP-dependent DNase, led to a lower DNA yield [32].

In summary, the results obtained in the present work show that these improvements in the general protocol for chloroplasts and cpDNA isolation in conifers enhance the overall quality and yield of chloroplasts and cpDNA isolation, providing a useful tool for studies that require isolated chloroplasts and/or plastid genome sequence. Facilitating chloroplast sequencing of this species group and, hence, increasing the amount of information about the plastid genome of conifers may, in turn, lead to greater understanding about plant evolution, as well as the structural and functional genomics in plants other than conifers.
